# Machine learning–based response assessment in patients with rectal cancer after neoadjuvant chemoradiotherapy: radiomics analysis for assessing tumor regression grade using T2-weighted magnetic resonance images

**DOI:** 10.1007/s00384-024-04651-6

**Published:** 2024-05-24

**Authors:** Yong Dae Lee, Hyug-Gi Kim, Miri Seo, Sung Kyoung Moon, Seong Jin Park, Myung-Won You

**Affiliations:** 1 Department of Radiology, Kyung Hee University Hospital, Kyung Hee University College of Medicine, #23 Kyungheedae-ro, Dongdaemun-gu, 02447 Seoul, Republic of Korea; 2 Department of Medicine, Kyung Hee University College of Medicine, 23 Kyungheedae-ro, Dongdaemun-gu, 02447 Seoul, Republic of Korea

**Keywords:** Rectal cancer, Treatment response assessment, Magnetic resonance imaging, Machine learning, Radiomics analysis, Tumor regression grade

## Abstract

**Purpose:**

This study aimed to assess tumor regression grade (TRG) in patients with rectal cancer after neoadjuvant chemoradiotherapy (NCRT) through a machine learning–based radiomics analysis using baseline T2-weighted magnetic resonance (MR) images.

**Materials and methods:**

In total, 148 patients with locally advanced rectal cancer(T2-4 or N+) who underwent MR imaging at baseline and after chemoradiotherapy between January 2010 and May 2021 were included. A region of interest for each tumor mass was drawn by a radiologist on oblique axial T2-weighted images, and main features were selected using principal component analysis after dimension reduction among 116 radiomics and three clinical features. Among eight learning models that were used for prediction model development, the model showing best performance was selected. Treatment responses were classified as either good or poor based on the MR-assessed TRG (mrTRG) and pathologic TRG (pTRG). The model performance was assessed using the area under the receiver operating curve (AUROC) to classify the response group.

**Results:**

Approximately 49% of the patients were in the good response (GR) group based on mrTRG (73/148) and 26.9% based on pTRG (28/104). The AUCs of clinical data, radiomics models, and combined radiomics with clinical data model for predicting mrTRG were 0.80 (95% confidence interval [CI] 0.73, 0.87), 0.74 (95% CI 0.66, 0.81), and 0.75(95% CI 0.68, 0.82), and those for predicting pTRG was 0.62 (95% CI 0.52, 0.71), 0.74 (95% CI 0.65, 0.82), and 0.79 (95% CI 0.71, 0.87).

**Conclusion:**

Radiomics combined with clinical data model using baseline T2-weighted MR images demonstrated feasible diagnostic performance in predicting both MR-assessed and pathologic treatment response in patients with rectal cancer after NCRT.

**Supplementary information:**

The online version contains supplementary material available at 10.1007/s00384-024-04651-6.

## Introduction

The standard treatment for locally advanced rectal cancer (LARC), defined as clinically determined T3/T4 or node-positive cancer, consists of neoadjuvant chemoradiotherapy (NCRT) or short-course preoperative radiotherapy followed by total mesorectal excision [1]. Response to neoadjuvant treatment correlates with long-term outcomes, and is a crucial factor in treatment planning in patients with LARC. Approximately 15–27% of patients with LARC achieve pathologic complete response (pCR) after chemoradiotherapy (CRT), which indicates no residual viable tumor cells in the resected specimen, and this pCR is associated with improved 5-year rates of local control, distant recurrence, disease-free survival (DFS), and overall survival (OS) [2, 3]. However, pCR can only be confirmed by pathologic examination of surgically resected specimens.

Response after CRT can be noninvasively assessed by rectal MRI using the mrTRG scoring, which is a 5-point grading system derived from pathologic tumor regression grade (pTRG) by the Magnetic Resonance Imaging and Rectal Cancer European Equivalence Study (MERCURY) group [4]. Previous studies have reported significant differences in DFS and OS between the good and poor response groups based on mrTRG [5, 6]. MRI-based good responders can be candidates for watch-and-wait (W&W) approach, and a previous multicenter registry study on W&W strategy revealed a 5-year disease specific survival of 94% [7]. Therefore, treatment response assessment using mrTRG could be a useful substitute for pTRG in preoperative decision-making and guidance for organ preservation strategy.

Radiomics involves the extraction of mineable, high-dimensional data from radiology images and has been applied to oncologic radiology to derive meaningful information on tumors from the extracted data [8]. Radiomics can provide information about the entire tumor phenotype and microenvironment that is difficult for radiologists to assess visually. Recent researches have conducted radiomics analyses of pre-treatment baseline MRI for response prediction [9, 10] and have mostly focused on pCR prediction [11–13]. In addition to pCR prediction, assessment and prediction of MRI-based treatment response would be important because of the increasing need to identify candidates for the organ-preserving strategy. Radiomics analysis can assist in the non-invasive prediction of preoperative treatment response, and be used as a screening tool to select candidates for the W&W strategy.

Therefore, this study aimed to investigate radiomics analysis and develop a machine-learning-based treatment response prediction model using T2-weighted images (T2WI) of baseline rectal MRI in patients with rectal cancer to identify good responders based on both mrTRG and pTRG.

## Materials and methods

### Patient selection and clinical data

**Fig. 1:**
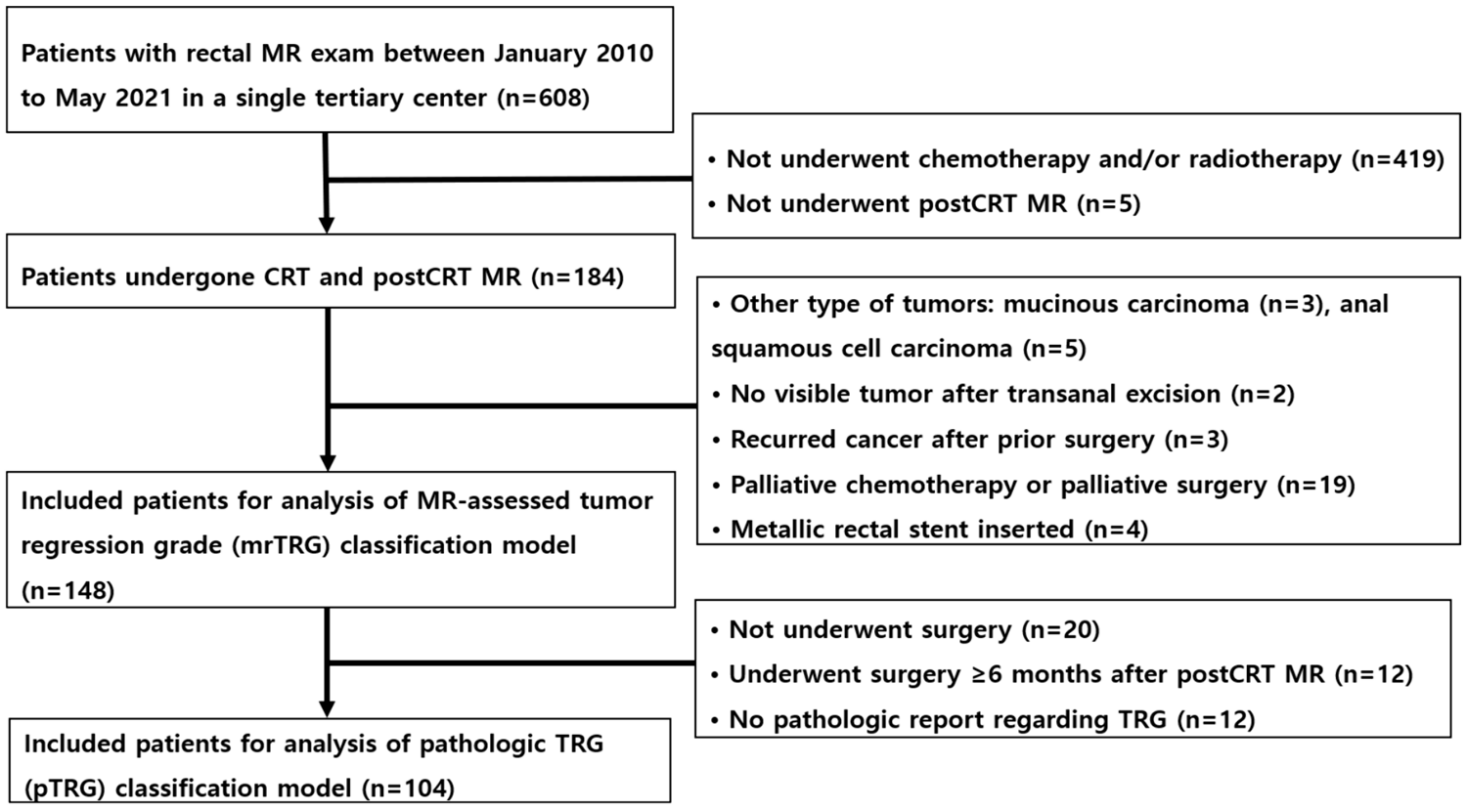
Patient selection

This retrospective study was approved by the institutional review board of Kyung Hee University Hospital (IRB No. 2023-12-023) and waived the requirement for informed consent. Patients who underwent baseline rectal MRI between January 2010 and May 2021 were enrolled consecutively. Patients with LARC (T2-4 or N+) who underwent NCRT and MRI after CRT at our institution were included in the study. The exclusion criteria were as follows: (1) did not receive neoadjuvant chemotherapy or/and radiotherapy (*n* = 418); (2) did not undergo post-CRT MRI (*n* = 5); (3)with other cancer types (*n* = 5, anal squamous cell carcinoma; *n* = 3, mucinous cancer); (4) without a visible tumor after transanal excision (*n* = 2); (5) received palliative chemotherapy or palliative surgery for distant metastasis (*n* = 19); (6) underwent metallic stent insertion (*n* = 4); (7) tumor recurrence after previous treatment (*n* = 3). Altogether, 148 patients were included for development of mrTRG prediction model. Among them, 104 patients were included in the development of pTRG prediction model after exclusion of patients: (1) who did not receive surgery (*n* = 20), (2) who received surgery > 6 months after post-CRT MRI because of the watch-and-wait approach (*n* = 12), and (3) who had no available pathologic reports regarding tumor regression grade (TRG) (*n* = 12). The patient selection process is illustrated in Fig. 1. Clinical data, including pathologic tumor differentiation grade from the initial endoscopic biopsy, mean CRT duration, mean interval between CRT and post-CRT MRI, mean interval between post-CRT MRI and surgery, and serum carcinoembryonic antigen (CEA) level at diagnosis (ng/mL, within 1 month of MRI), were recorded.

This study followed the Standards for Reporting Diagnostic accuracy studies (STARD) reporting guidelines [14].

### Magnetic resonance (MR) image acquisition and protocol

Baseline MR images were acquired with Achieva 3T (Philips Healthcare system, 134/148; 90.5%) and MAGNETOM Vida 3T (Siemens Healthineers System, 10/148; 6.7%) using a 32-ch coil. High-spatial-resolution axial oblique T2WI, which were obtained perpendicular to the long axis of the tumor and/or area after treatment, were used for both visual assessment and radiomics analysis. Diffusion-weighted imaging (DWI) (*b*-value = 0.1400) with an apparent diffusion coefficient (ADC) map was used when necessary. The MR parameters at our institution are presented in Supplementary Table 1.

### Radiomics analysis

Image segmentation: One radiologist (M. W. Y. with 15 years of experience in rectal imaging) manually segmented the entire tumor mass and area after treatment within the rectal wall on each slice of oblique axial T2WI of baseline and post-CRT MR exams using the MEDIP Pro software (v2.0.0.0, MEDICALIP Co. Ltd., Seoul, Korea). The radiologist was blinded to histopathologic data and treatment outcomes, except for the diagnosis of rectal cancer and post-CRT status. Region of interest (ROI) was drawn along the margin of the tumor signal not to include outer non-rectal tissue and normal rectum on baseline MR. For segmentation on post-CRT MRI, baseline MRI was used to identify initial tumor extent, and a ROI was drawn covering the entire area of tumor bed including signal intensity demonstrating fibrosis or mucin.

Radiomics feature extraction and creation of dataset: All radiomic features, including volume, shape, intensity, and texture, were extracted from the original image. Features were extracted for T2WI using the MEDIP Pro software based on PyRadiomics texture measures comprising 2D size and shape-based features (*n* = 9), 3D size and shape-based features (*n* = 14), histogram-based first-order statistical features (*n* = 18), second-order statistical features, and gray-level co-occurrence matrix features regarding the relationships between image voxels (*n* = 75) [15]. To create dataset for machine learning model, the extracted feature values with 116 and three clinical factors composed of serum CEA, tumor differentiation grade, and cT stage were used. The datasets were constructed to six types: clinical factors only, radiomics features of baseline MR, radiomics features with serum CEA, radiomics features with cT stage, radiomics features with tumor differentiation grade, and combined radiomics features with three clinical factors.

Data processing and dimension reduction: Before training the model, data preprocessing was performed to normalize the original data with the various range of the data value. The range of data value was adjusted to the values within the range of 0 to 1 using min-max scaling. In addition, principal component analysis (PCA) was performed on the scaled data to reduce dimensionality, because relatively small study population requires the reduction in the number of selected features to improve model performance. Six case datasets combined with 116 significant features and three clinical factors were refiltered using the Python model and PCA, respectively.

**Fig. 2:**
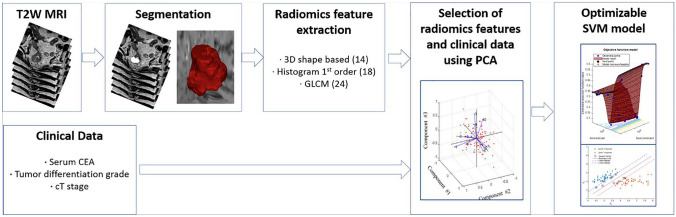
Radiomics analysis and model development

**Fig. 3:**
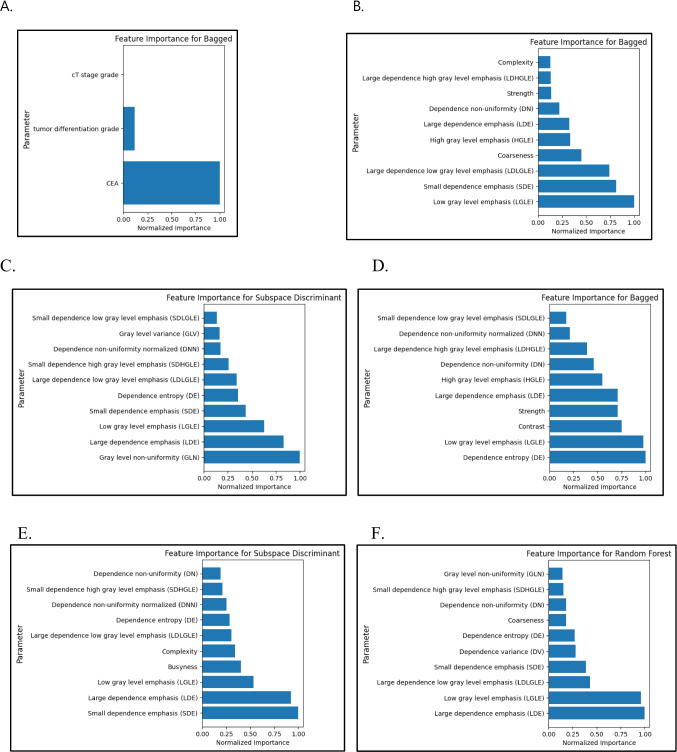
Feature importance plots for six models. **A** Clinical data model. **B** Radiomics model. **C** Radiomics with serum CEA model. **D** Radiomics with cT stage model. **E** Radiomics with tumor differentiation grade model. **F** Combined radiomics with all clinical data model. Horizontal axis represents feature weights

Classification model building for treatment outcomes: Grid search was conducted to identify a suitable model for the extracted features and determine the hyperparameter tuning condition for optimal training in binary classification. In this process, we assessed the generalization ability of the model through a threefold cross validation. The ensemble learning algorithms comprised boosted, bagged, subspace discriminant, subspace KNN, RUSBoost trees, random forest, logistic regression, and support vector machines (SVM) in our model (Table 1). Based on the grid-search results, we selected an optimal condition for each dataset. The total dataset was randomly divided into a training dataset for model training and a test dataset for model validation in a 7:3 ratio, with an equal ratio of the two response groups between the two data sets. In our study, the training process was conducted in a Python environment, and Python libraries were utilized to evaluate the performance, such as area under the receiver operating characteristic curve (AUROC), AUC score, accuracy sensitivity, specificity, positive predictive value (PPV), and negative predictive value (NPV) (Fig. 2). Furthermore, we confirmed feature importance for each dataset to observe the characteristics influencing training results (Fig. 3).

**Table 1:** Selected feature parameters and used learning models

Dataset	Number of parameters	Number of main features^a^	Learning models
Clinical data: serum CEA, tumor diff.grade, T stage	3	3	SVM Bagged
Radiomics of baseline MR	116	18	Boosted
Radiomics + serum CEA	117 (116 + 1)	18	Random forest
Radiomics + cT stage	117 (116 + 1)	19	RUSBoost trees
Radiomics + tumor diff. grade	117 (116 + 1)	19	Subspace KNN Logistic Regression
Radiomics + all three clinical factors	119 (116 + 3)	20	Subspace Discriminant

^a^Selected features after dimension reduction using principal component analysis

### MR-assessed and pathologic tumor response

Two radiologists (with 15 and 25 years of experience in rectal imaging, respectively) reviewed the baseline and post-CRT T2WI and DWI with ADC maps in consensus. The reviewers were blinded to the clinical and pathological data, except for the post-CRT status of the rectal adenocarcinoma. mrTRG was assigned according to a five-point system [16] (Supplementary Table 2). mrTRG 1–3 and mrTRG 4–5 were classified as having good and poor responses, respectively, according to previous reports [6, 17]. The radiologists used baseline MR images to guide the delimitation of the tumor bed, and DWI with ADC map was additionally assessed when the reviewers were uncertain regarding the presence of a viable tumor. Another reviewer (with 18 years of experience in rectal imaging) determined T stage on baseline MR T2WI according to American Joint Committee on Cancer 8th edition [18], blinded to histopathologic data and treatment outcome. Clinical T stage was classified into T2/T3a, T3b/T3c/T3d, and T4a/T4b to increase the number of cases in each category and enhance comparability between the groups during classification model building. Interobserver agreement between the two reviewers were assessed additionally after more than 1 month of wash-out period.

The pathological treatment response was determined according to the reported Dworak TRG system [19] (Supplementary Table 2), based on the results of the surgical pathological analysis. pTRG 3–4 and pTRG 0–2 were classified as having good and poor responses, respectively, according to previous reports [20, 21].

### Statistical analyses

Categorical data are presented as percentages, and numerical data are presented as means with standard deviations or medians with interquartile ranges (IQR), as appropriate. Indeterminate or missing mrTRG data were not included in the radiomics analysis. The model performance for predicting good or poor response based on mrTRG and pTRG were assessed using an AUROC analysis, and diagnostic performance of each model was determined by sensitivity, specificity, accuracy, PPV, and NPV. For each six model for either mrTRG or pTRG, AUC results of eight learning models were compared, and the best performance for each six dataset among them was selected. Interobserver agreement were evaluated using Cohen’s kappa coefficient: 0.1–0.20 slight, 0.21–0.40 fair, 0.41–0.60 moderate, 0.61–0.80 substantial, and 0.81–0.99 near perfect. Statistical analyses were performed using Python 3.9.0 and Medcalc (ver. 20.111 Medcalc software Ltd., Ostend, Belgium), and significance was set at *P* < .05.

## Results

### Patient characteristics

The mean age of included patients was 62.8 ± 11.6, and male patients comprised 67.6% (100/148) of the included patients. The median CRT duration was 42 days (IQR 38–46 days), the median interval between CRT and post-CRT MR was 47.5 days (IQR 30–56), the median interval between post-CRT MR and surgery was 12 days (IQR 6–27.25), and the median serum CEA was 4.56 ng/ml (IQR 2.52–12.98). Clinical T stage was mostly T3b/c/d (69.6%, 103/148), and pathological tumor grade was mostly moderately differentiated adenocarcinoma (75%, 111/148). Clinical N stage was mostly N2 (42.5%, 63/148) followed by N1 (39.2%, 58/148) and N0 (16.9%, 25/148). According to mrTRG grading, the good response group comprised 49.3% (73/148), and the poor response group comprised 50.7% (75/148) of the patients. Meanwhile, according to pTRG grading, the good response group comprised 26.9% (28/104), and the poor response group comprised 73% (76/104) of the patients. Patient characteristics are summarized in Table 2.

**Table 2:** Clinical data of included patients

	*N* (= 148)
Age at diagnosis (years, mean ± SD)	62.8 (± 11.6)
Male (*n*, %)	100 (67.6)
CRT duration (d, median, IQR)	42 (38–46)
Interval between CRT and post-CRT MR (d, median, IQR)	48 (30–56)
Interval between post-CRT MR and surgery (d, median, IQR)	13 (6–29)
Surgery (*n*, %)	127 (85.8)
Serum CEA at diagnosis (ng/ml, median, IQR)	4.56 (2.52–12.98)
Clinical T stage (*n*,%)	
1: T2, T3a	16 (10.8)
2: T3b, T3c, T3d	103 (69.6)
3: T4a, T4b	27 (18.2)
Clinical N stage (*n*,%)	
N0	25 (16.9)
N1	58 (39.2)
N2	63 (42.5)
Pathologic tumor grade (*n*,%)	
Well differentiated	26 (17.6)
Moderately differentiated	111 (75)
Poorly differentiated	8 (5.4
Treatment response on MR exam (*n*, %)	
Good response: mrTRG 1/mrTRG 2/mrTRG 3	73 (49.3): 3 (2)/20 (13.5)/50 (33.8)
Poor response: mrTRG 4/mrTRG 5	75 (50.7):72 (48.6)/3 (2)
Pathologic treatment response (Dworak, *n*,%)	*N* = 104
Good response: TRG 3 (near complete)/TRG 4 (total regression)	28 (26.9): 13 (12.5)/15 (14.4)
Poor response: TRG 0 (no)/TRG 1 (minimal)/TRG 2 (moderate)	76 (73): 3 (2.8)/33 (31.7)/40 (38.5)

*SD* standard deviation, *SCC* squamous cell carcinoma, *CRT* chemoradiation therapy, *d* days, *CEA* carcino-embryonic antigen, *TRG* tumor regression grade

### Diagnostic performance of radiomics analyses for the classification of response groups

**Fig. 4:**
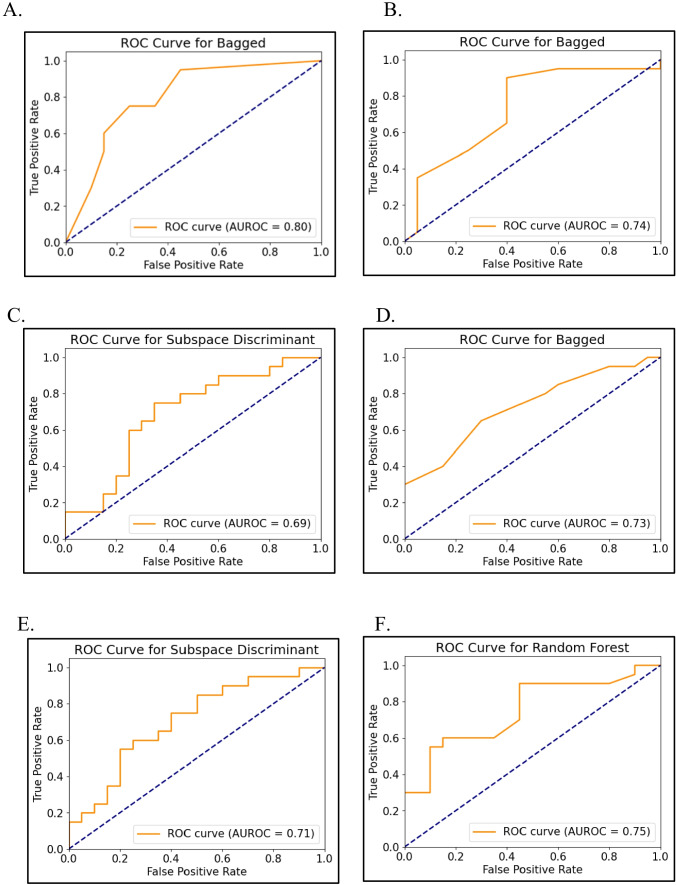
Predictive performance of the machine learning models in differentiating good (mrTRG 1–3) and poor (mrTRG 4–5) response group. **A** Clinical data model. **B** Radiomics model. **C** Radiomics with serum CEA model. **D** Radiomics with cT stage model. **E** Radiomics with tumor differentiation grade model. **F** Combined radiomics with all clinical data model

**Fig. 5:**
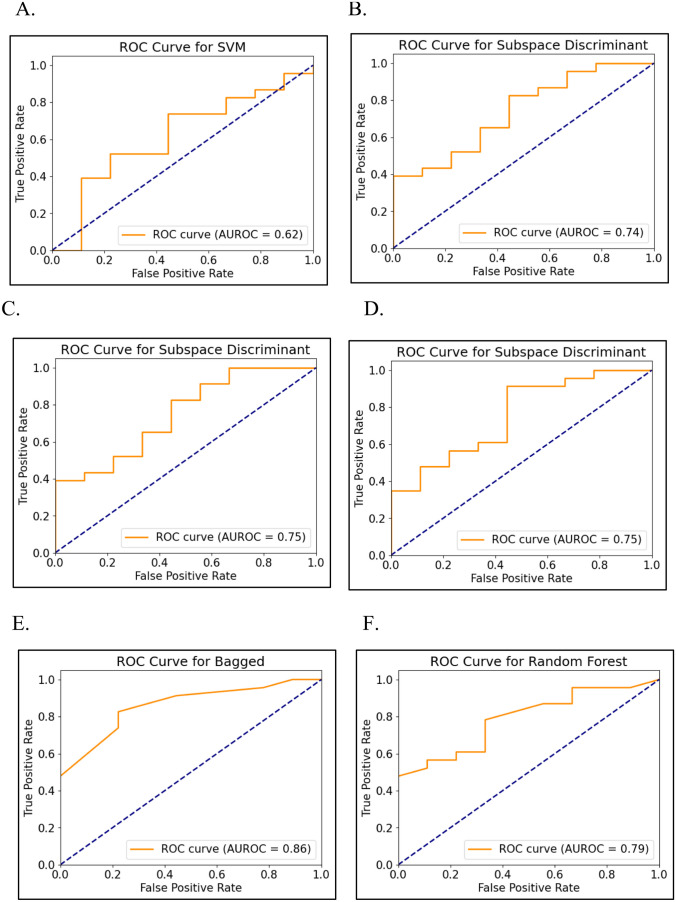
Predictive performance of the machine learning models in differentiating good (pTRG 3–4) and poor(pTRG 0–2) response group. **A** Clinical data model. **B** Radiomics model. **C** Radiomics with serum CEA model. **D** Radiomics with cT stage model. **E** Radiomics with tumor differentiation grade model. **F** Combined radiomics with all clinical data model

The interobserver agreement of the mrTRG was near perfect (*k*-value 0.81, 95% CI 0.73–0.89). The ROC curve analyses of machine learning models using baseline MR radiomics features and clinical data for the prediction of treatment response based on mrTRG are presented in Table 3 and Fig. 4, and pTRG in Table 4 and Fig. 5, respectively. Among six models for predicting good or poor response based on mrTRG, the clinical data model, radiomics model, and combined radiomics with all the clinical data model showed the best AUCs of 0.80 (95% CI 0.73–0.87, sensitivity 75%, specificity 75%, accuracy 75%), 0.74 (95% CI 0.66–0.81, sensitivity 65%, specificity 60%, accuracy 62%), and 0.75 (95% CI 0.68–0.82, sensitivity 70%, specificity 55%, accuracy 62%) respectively, whereas the radiomics with one clinical factor model demonstrated a slightly lower AUC of 0.69–0.73.

**Table 3:** Performance of six clinical, radiomics, and combined models in the differentiating good and poor response groups based on mrTRG

Dataset	Model	Radscore						
		AUC(95% CI)	SD-AUC	Accuracy	Sensitivity	Specificity	PPV	NPV
Clinical data: serum CEA, tumor diff.grade, T stage	Bagged	0.80 (0.73, 0.87)	0.80	0.75	0.75	0.75	0.75	0.75
	Subspace KNN	0.78 (0.71, 0.85)	0.41	0.72	0.80	0.65	0.65	0.76
	Random Forest	0.78 (0.71, 0.85)	0.42	0.72	0.75	0.70	0.70	0.74
	Boosted	0.73 (0.65, 0.80)	0.44	0.72	0.80	0.65	0.65	0.76
Radiomics of baseline MR	Bagged	0.74 (0.66, 0.81)	0.44	0.62	0.65	0.60	0.62	0.63
	Subspace discriminant	0.68 (0.60, 0.76)	0.47	0.62	0.75	0.50	0.60	0.67
	Logistic regression	0.67 (0.59, 0.75)	0.47	0.60	0.65	0.55	0.59	0.61
	RUSBoost Trees	0.64 (0.56, 0.73)	0.48	0.60	0.65	0.55	0.59	0.61
Radiomics + serum CEA	Subspace discriminant	0.69 (0.61, 0.77)	0.46	0.68	0.80	0.55	0.64	0.73
	Logistic regression	0.67 (0.59, 0.75)	0.47	0.65	0.75	0.55	0.62	0.69
	Random forest	0.66 (0.58, 0.75)	0.47	0.62	0.60	0.65	0.63	0.62
	Bagged	0.64 (0.55, 0.72)	0.48	0.52	0.60	0.45	0.52	0.53
Radiomics + cT stage	Bagged	0.73 (0.62, 0.80)	0.45	0.68	0.65	0.70	0.68	0.67
	Subspace discriminant	0.72 (0.64, 0.79)	0.45	0.68	0.80	0.55	0.64	0.73
	Logistic regression	0.70 (0.62, 0.78)	0.46	0.65	0.75	0.55	0.62	0.69
	RUSBoost Trees	0.69 (0.61, 0.77)	0.46	0.65	0.65	0.65	0.65	0.65
Radiomics + tumor diff. grade	Subspace discriminant	0.71 (0.62, 0.78)	0.45	0.68	0.75	0.60	0.65	0.71
	Random forest	0.70 (0.62, 0.78)	0.46	0.70	0.65	0.75	0.72	0.68
	Logistic regression	0.68 (0.60, 0.75)	0.47	0.65	0.75	0.55	0.62	0.69
	Subspace KNN	0.63 (0.55, 0.71)	0.48	0.60	0.65	0.55	0.59	0.61
Radiomics + all three clinical factors	Random forest	0.75 (0.68, 0.82)	0.43	0.62	0.70	0.55	0.61	0.65
	Subspace discriminant	0.73 (0.66, 0.81)	0.44	0.70	0.75	0.65	0.68	0.72
	Logistic regression	0.70 (0.63, 0.78)	0.46	0.62	0.75	0.50	0.60	0.67
	Bagged	0.66 (0.58, 0.74)	0.47	0.60	0.45	0.75	0.64	0.58

*AUC* area under the curve, *SD-AUC* standard deviation of AUC, *PPV* positive predictive value, *NPV* negative predictive value

**Table 4:** Performance of six clinical, radiomics, and combined models in the differentiating good and poor response groups based on pTRG

Dataset	Model	Radscore						
		AUC(95% CI)	SD-AUC	Accuracy	Sensitivity	Specificity	PPV	NPV
Clinical data: serum CEA, tumor diff.grade, T stage	SVM	0.62 (0.52, 0.71)	0.49	0.72	1.00	0.00	0.72	0.00
	Logistic regression	0.48 (0.38, 0.57)	0.50	0.72	1.00	0.00	0.72	0.00
	Subspace discriminant	0.43 (0.33, 0.52)	0.49	0.72	1.00	0.00	0.72	0.00
	Bagged	0.33 (0.24, 0.42)	0.47	0.50	0.70	0.00	0.64	0.00
Radiomics of baseline MR	Subspace discriminant	0.74 (0.65, 0.82)	0.44	0.75	0.83	0.56	0.83	0.56
	Logistic regression	0.71 (0.62, 0.79)	0.46	0.75	1.00	0.11	0.74	1.00
	Bagged	0.67 (0.58, 0.76)	0.47	0.69	0.83	0.33	0.76	0.43
	Random forest	0.63 (0.54, 0.73)	0.48	0.69	0.91	0.11	0.72	0.33
Radiomics + serum CEA	Subspace discriminant	0.75 (0.67, 0.83)	0.43	0.75	0.83	0.56	0.83	0.56
	Random forest	0.71 (0.62, 0.79)	0.46	0.75	1.00	0.11	0.74	1.00
	Logistic regression	0.70 (0.61, 0.79)	0.46	0.75	1.00	0.11	0.74	1.00
	Boosted	0.64 (0.55, 0.73)	0.48	0.81	0.96	0.44	0.81	0.80
Radiomics + cT stage	RUSBoost trees	0.77 (0.69, 0.85)	0.42	0.75	0.83	0.56	0.83	0.56
	Random forest	0.75 (0.67, 0.84)	0.43	0.78	1.00	0.22	0.77	1.00
	Subspace discriminant	0.75 (0.67, 0.84)	0.43	0.75	0.83	0.56	0.83	0.56
	Logistic regression	0.73 (0.64, 0.81)	0.44	0.75	1.00	0.11	0.74	1.00
Radiomics + tumor diff. grade	Bagged	0.86 (0.79, 0.93)	0.35	0.81	0.83	0.78	0.90	0.64
	Random forest	0.85 (0.78, 0.92)	0.35	0.75	1.00	0.11	0.74	1.00
	Logistic regression	0.72 (0.63, 0.81)	0.45	0.75	1.00	0.11	0.74	1.00
	Subspace discriminant	0.71 (0.63, 0.80)	0.45	0.75	0.83	0.56	0.83	0.56
Radiomics + all three clinical factors	Random forest	0.79 (0.71, 0.87)	0.41	0.75	0.96	0.22	0.76	0.67
	Boosted	0.75 (0.67, 0.83)	0.43	0.66	0.87	0.11	0.71	0.25
	Subspace discriminant	0.75 (0.67, 0.83)	0.43	0.75	0.83	0.56	0.83	0.56
	Logistic regression	0.71 (0.63, 0.80)	0.45	0.75	1.00	0.11	0.74	1.00

*AUC* area under the curve, *SD-AUC* standard deviation of AUC, *PPV* positive predictive value, *NPV* negative predictive value

**Fig. 6:**
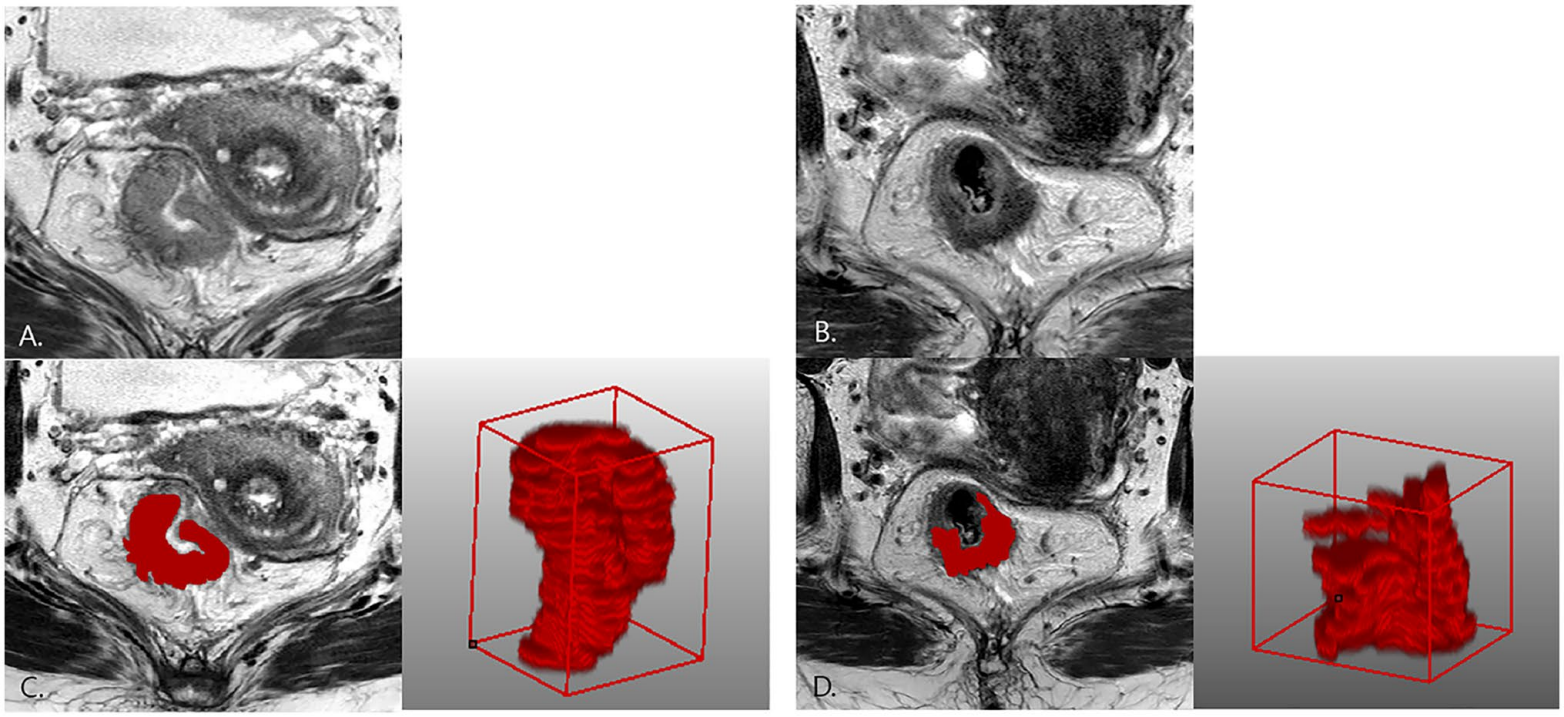
A 53-year-old female patient in good response group in both mrTRG and pTRG. Serum CEA was 7.79 ng/ml, clinical T stage was T3c, and moderately differentiated adenocarcinoma at baseline. mrTRG was graded as 3, and Dworak pTRG was grade 4 (total regression, ypT0). **A** Baseline T2W oblique axial image shows intermediate SI tumor mass circumferentially encircling lumen sparing left anterior wall only. **B** PostCRT MR image shows decreased volume and SI of tumor mass. Predominent fibrosis was mixed with some intermediate SI portion; therefore, mrTRG was graded as 3. **C**, **D** ROI was drawn at baseline and postCRT tumor, and the 3D models were generated. mrTRG classification based on combined radiomics with clinical data model was good response, and pTRG classification based on radiomics, and combined radiomics with clinical data model was good response, which were concordant with mrTRG and pTRG

**Fig. 7:**
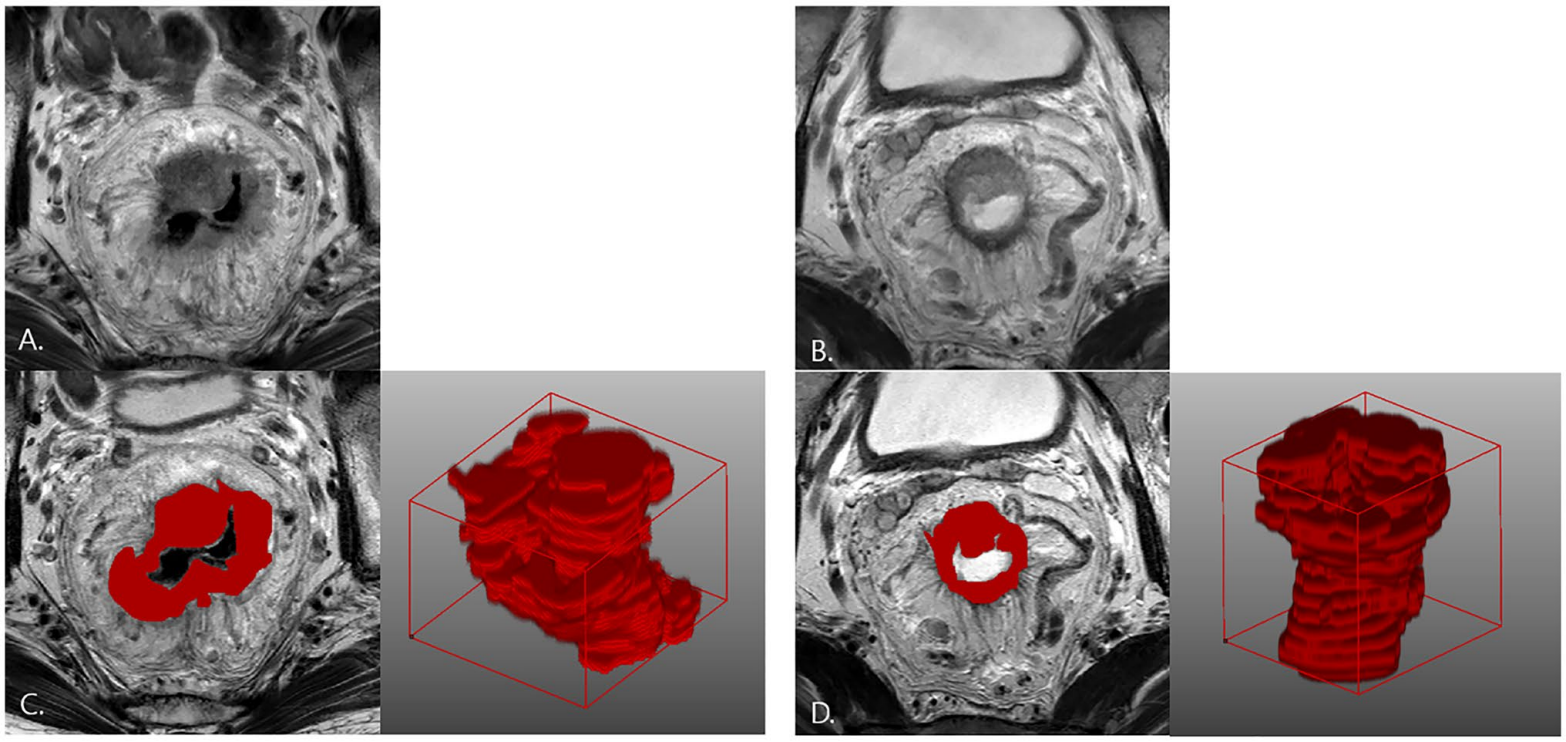
A 46-year-old male patient in poor response group. Serum CEA was 15.34 ng/ml, clinical T stage was T3c and moderately differentiated adenocarcinoma at baseline. mrTRG was graded as 4, and Dworak pTRG was grade 1 (minimal regression, ypT3). **A** Irregular-shaped circumferentially encircling mass at baseline T2W oblique axial image. **B** postCRT MR shows decreased volume and SI of cancer mass; however, considerable remnant tumor was exist; therefore, mrTRG was graded as 4. **C**, **D** ROI was drawn at baseline and postCRT tumor mass, and the 3D models were generated. mrTRG classification based on radiomics and combined radiomics with clinical data was poor response, and pTRG classification based on combined radiomics with clinical data model was poor response, which were concordant with mrTRG and pTRG

For another six models for predicting treatment response based on pTRG, combined radiomics with all the three clinical data model demonstrated the highest best AUC (0.79, 95% CI 0.71–0.87, sensitivity 96%, specificity 22%, accuracy 75%) followed by the radiomics with tumor differentiation grade model (AUC 0.86; 95% CI 0.79–0.93, sensitivity 83%, specificity 78%, accuracy 81%) and the radiomics with cT stage model (AUC 0.77; 95% CI 0.69–0.85, sensitivity 83%, specificity 78%, accuracy 81%). The radiomics model demonstrated similar best AUC to that based on mrTRG (AUC 0.74; 95% CI 0.65–0.82). Representative cases in the good and poor response groups are presented in Figs. 6 and 7, respectively.

## Discussion

In our study, we evaluated a machine learning–based classification model using baseline MR radiomics for both mTRG and pTRG prediction, and the radiomics model demonstrated similar best classification performance for good or poor response between mrTRG-based and pTRG-based prediction (AUC 0.74). Moreover, combined radiomics with clinical data model presented enhanced best classification performance for both pTRG-based (0.79) and mrTRG-based response prediction (0.75) compared with those of radiomics only model. Recent studies on treatment response prediction using baseline MR radiomics mostly investigated the prediction of pCR [12, 22–24] or pTRG [25–27], and favorable model performances ranging between 0.75 and 0.84 have been reported. Delli Pizza et al. have reported similar results to ours, that is, superior performance of a combined MR radiomics and clinical features model compared with clinical features or radiomics-only models (0.79 vs. 0.68 and 0.7) [26]. Although the clinical data component for model development was simpler than our study (serum CEA, T stage, and pathologic tumor grade), they also used solely T2WI for radiomics analysis and demonstrated enhanced model performance for combined clinical and radiomics data as well. As serum CEA levels can be a simple and good predictor of post-CRT treatment response [28, 29], we recommend including serum CEA level as a clinical factor. Several studies have investigated multiparametric MR radiomics using not only T2WI but also T1 contrast-enhanced and DWI [12, 22, 24]. However, we used only baseline T2WI for the radiomics analysis to reduce the feature dimension and analysis burden, thereby maintaining model performance with the present study population because increased feature dimension with additional DWI images may require a larger study population. T2WIs are key images for MR tumor evaluation and more appropriate for contrast and texture analysis; therefore, we investigated T2WI-based radiomics analysis by priority.

Research on radiomics for mTRG prediction has been limited. Van Griethuysen et al. developed a radiomics model using baseline MR for MR-assessed response prediction and reported AUCs of 0.69–0.79 [30]. However, they developed their own MR-assessed response criteria that comprised several morphological features and tumor-node stages. We used the mrTRG system, which was developed by the MERCURY group and is widely used for treatment response assessments worldwide, including in our institution. MR-assessed TRG is a useful surrogate marker for pathologic TRG for response assessment because it can be used without or before surgery; therefore, it can guide treatment follow-up strategies and select candidates for organ preservation. Hence, we investigated the radiomics prediction model for mrTRG and observed a similar performance to that of pTRG. Therefore, our results might broaden the field of MR radiomics analysis and contribute to preoperative and noninvasive guidance of treatment planning in patients with post-CRT rectal cancer.

We established good-response groups that were slightly different between the mrTRG and pTRG groups. For mrTRG, mrTRG 1–3 was defined as a good response, including the gray zone in the good-response group. Several studies defined a good response as mrTRG 1–3 [5, 6, 17], whereas others defined a good response as mrTRG 1–2 [31, 32]. We defined the good response group as a wider range, including mrTRG 3, to build a screening model for selecting candidate for watch-and-wait approach. This screening approach can assist radiologists and referring physicians in making decisions for patients.

During the training process of radiomics analysis, we used ensemble method comprising eight kinds of models including boosted, bagged, random forest, logistic regression, RUSBoost trees, subspace KNN, subspace discriminant, and SVM for model building. The hold-out method was utilized to check split dataset with train/test of 7:3 ratio, and threefold cross-validation method was used for all data validation process. These techniques are proper to reduce the effect of limited input data and increase the validation reliability [33].

Our study has several limitations. First, this was a single-center retrospective study, which may indicate a potential selection bias and limited generalizability. Second, MR images were acquired from two different vendors (Philips and Siemens); however, both were 3T, and all MR images were obtained with the same slice thickness (3 mm). Furthermore, the extracted radiomic features were scaled using min–max scaling prior to training. Third, not all patients underwent surgical resection, although we aimed to evaluate the preoperative and non-invasive prediction of post-CRT response; therefore, mTRG was the final endpoint of the classification model, as well as pTRG in the subgroup that received surgery. Fourth, we did not validate an outer cohort; however, we evaluated the model performance with the test set separated from the training set during model development. Our model performance was evaluated using the unseen test set data. Lastly, we did not consider sequences other than T2W images, such as DWI or ADC, for the radiomics analysis. We focused on T2W image analysis, which is the most important sequence for tumor evaluation on rectal MR.. Further work with additional sequences would improve model performance.

In conclusion, the machine learning–based radiomics model using baseline T2WI, especially when combined with clinical data, demonstrated favorable performance in predicting good or poor response after CRT in patients with rectal cancer. This combined clinical and radiomics data model was useful for predicting treatment response based on both mrTRG and pTRG, thereby supporting communication with patients or referring physicians, screening candidates for organ preservation, and frequent and meticulous follow-up during treatment.

## Supplementary information

Below is the link to the electronic supplementary material.

Supplementary file1 (DOCX 17.9 kb)

## Data Availability

The datasets generated or analyzed during the study are available from the corresponding author on reasonable request.
