# Supplementary material for: Machine learning–based response assessment in patients with rectal cancer after neoadjuvant chemoradiotherapy: radiomics analysis for assessing tumor regression grade using T2-weighted magnetic resonance images
Source: Int J Colorectal Dis. 2024 May 24;39(1):78. doi: 10.1007/s00384-024-04651-6 (PMC11126485; doi:10.1007/s00384-024-04651-6)
Supplement: Supplementary file 1 — Supplementary file1 (DOCX 17.9 kb) [file 384_2024_4651_MOESM1_ESM.docx]

Supplementary Table 1. MR imaging parameters

|  | sequence | Repetition time(msec)/echo time (msec) | matrix | Field of view (mm) | Section thickness/ gap (mm) | Bandwidth (kHz)  /FlipAngle(degree) | b value  (sec/mm^2^) |
| --- | --- | --- | --- | --- | --- | --- | --- |
| Oblique axial T2W imaging  Achieva 3T  Vida 3T | TSE  TSE | 3200-5800/90  4300/102 | 392x254  352x246 | 160  160 | 3/0  3/0.3 | 0.218/90  0.268/120 |  |
| DWI  Achieva 3T  Vida 3T | DWI  DWI | 5000-8000/80  7900/76 | 127x125  120x120 | 220  220 | 4/1  3/0 | 2.225/90  1.603/90 | 1000  1000 |

T2W=T2-weighted, DWI= diffusion-weighted imaging, TSE= turbo spin-echo

Supplementary Table 2. MR and pathologic tumor regression grade systems

1) MR tumor regression grade (mrTRG)

| **Grade** | **Response** | **MR Finding** |
| --- | --- | --- |
| mrTRG 1 | Complete response | Linear/crescentic 1–2 mm scar in mucosa or submucosa only |
| mrTRG 2 | Good response | Dense fibrosis; no obvious residual tumor, signifying minimal residual disease or no tumor |
| mrTRG 3 | Moderate response | > 50% fibrosis/mucin and visible intermediate signal |
| mrTRG 4 | Slight response | Little areas of fibrosis or mucin, but mostly tumor |
| mrTRG 5 | No response | Intermediate signal intensity, same appearances as original tumor/tumor regrowth |

2) Pathologic Dworak tumor regression grade (pTRG)

| **Grade** | **Response** | **Finding** |
| --- | --- | --- |
| TRG 0 | No regression | No regression |
| TRG 1 | Minimal regression | Dominant tumor mass with obvious fibrosis |
| TRG 2 | Moderate regression | Dominant fibrotic changes with few tumor cells or groups |
| TRG 3 | Near complete regression | Very few tumor cells |
| TRG 4 | Complete regression | No tumor cells |
